# Extracellular Volume Fraction Combined With Pathological Features of α‐SMA and FAP for Predicting the Prognosis of Patients With Pancreatic Ductal Adenocarcinoma After Surgery and Evaluating the Efficacy of Chemotherapy

**DOI:** 10.1002/cam4.71281

**Published:** 2025-10-02

**Authors:** Liqiang Liu, Sicheng Xu, Pengbiao Miao, Xing He, Yaorong Peng, Qixian Zhou, Junkai Ren, Zhenyu Zhou, Huilin Ye, Wenbin Li

**Affiliations:** ^1^ Department of Biliary and Pancreatic Surgery, Sun Yat‐Sen Memorial Hospital Sun Yat‐Sen University Guangzhou P.R. China; ^2^ Guangdong Provincial Key Laboratory of Malignant Tumor Epigenetics and Gene Regulation, Sun Yat‐Sen Memorial Hospital Sun Yat‐Sen University Guangzhou P.R. China; ^3^ Department of Hepatobiliary Surgery, Sun Yat‐Sen Memorial Hospital Sun Yat‐Sen University Guangzhou P.R. China

**Keywords:** computed tomography, extracellular volume fraction, pancreatic ductal carcinoma, prognosis, tumor microenvironment

## Abstract

**Objectives:**

This study aimed to evaluate the extracellular matrix of pancreatic ductal adenocarcinoma (PDAC) using the extracellular volume fraction (fECV) derived from enhanced CT images, integrating fECV with α‐SMA‐positive cancer‐associated fibroblasts (CAFs) and FAP‐positive CAFs to investigate their relationship with clinicopathological characteristics and prognosis of patients with PDAC.

**Methods:**

A retrospective analysis of 124 patients who underwent surgical resection for PDAC was conducted. Immunohistochemistry was applied to determine the expression of α‐SMA and FAP. fECV was calculated by attenuation values of PDAC and aorta. The Kaplan–Meier method was used to plot the postoperative overall survival (OS) and disease‐free survival (DFS) curves. A Cox proportional hazards regression model was used to develop a predictive model.

**Results:**

High α‐SMA (OS: *p* < 0.001; DFS: *p* = 0.065) and FAP (OS: *p* < 0.001; DFS: *p* < 0.001) expressions and low fECV (OS: *p* < 0.001; DFS: *p* < 0.001) predict poor prognosis. Patients with co‐high expression of α‐SMA and FAP had worse OS and DFS. Multivariable analysis identified α‐SMA (OS: hazard ratio [HR], 2.34 [95% CI, 1.30–4.21], *p* = 0.005), FAP (OS: HR, 4.43 [95% CI, 2.72–7.19], *p* < 0.001), and fECV (OS: HR, 0.58 [95% CI, 0.37–0.90], *p* = 0.015) as independent predictors of prognosis. The predictive model established by combining fECV with α‐SMA and FAP in this study cohort demonstrated the best predictive value.

**Conclusions:**

The integration of fECV with α‐SMA and FAP expressions offers a robust method for predicting clinical outcomes in PDAC patients, potentially guiding treatment strategies.

## 1 Introduction

The 2024 US statistics reported a poor 5‐year survival rate of merely 13%, making pancreatic ductal adenocarcinoma (PDAC) the lowest surviving malignant tumor [1]. Given the early onset of the disease, approximately 80%–85% of patients with PDAC are diagnosed with locally advanced or distant metastases [2]. Even patients with locally resectable tumors have a poor prognosis, with a 5‐year survival rate of only 20%.

PDAC tumor microenvironment (TME) is characterized by abundant populations of cancer‐associated fibroblasts (CAFs), leading to extensive and dense proliferation of the connective tissue. The barrier formed by extracellular matrix (ECM) secreted by CAFs is a major factor contributing to the limited diffusion of chemotherapy drugs [3, 4]. A dense and poorly vascularized matrix damages the tumor vascular system, hindering drug penetration and inducing hypoxia. This environment promotes tumor invasion and resistance to treatment [5].

Myofibroblasts (myCAFs) are associated with TME remodeling [6], indicated by elevated α‐smooth muscle actin (α‐SMA) expression, secrete collagen, fibronectin, hyaluronic acid, and elastin, thereby contributing to dense ECM formation [7, 8], resulting in insufficient blood perfusion, leading to poor diffusion of drugs such as gemcitabine that causes PDAC to acquire chemotherapy resistance [4]. Patients with high α‐SMA expression levels have been correlated with notably worse outcomes [9]. Fibroblast activation protein (FAP) is selectively expressed in CAFs. FAP‐positive CAFs are associated with connective tissue hyperplasia and poor overall survival (OS) outcomes [10, 11, 12]. Therefore, the complex fibrotic microenvironment of PDAC is the reason for the poor prognosis and presents challenges in developing effective treatment strategies. Because the TME of PDAC contains various subtypes of CAFs, assessing the abundance of specific CAF populations through pathology does not accurately represent the entire microenvironment. Imaging examinations can facilitate the evaluation of the overall characteristics of the microenvironment.

The TME is reflected by the extracellular volume. During enhanced computed tomography (CT) examination, iodine contrast agents can freely pass through the vascular lumen but cannot enter the intracellular space [13, 14]. Therefore, by comparing the changes in attenuation values between contrast‐enhanced and non‐enhanced CT images of PDAC lesions and the abdominal aorta, the extracellular volume fraction (fECV) can be calculated. The fECV not only represents the extracellular space composed of extracellular components and vascular structures [13] but also reflects the degree of lesion vascularity. Therefore, fECV gradually increases with the progression of fibrosis and decreases with a reduction in vascular content. Currently, fECV is utilized to assess fibrosis in the heart, liver, and pancreas [13, 14, 15]. The fECV serves as an independent predictor of OS and progression‐free survival (PFS) in patients with stage IV pancreatic cancer undergoing chemotherapy [16] and has a predictive value in evaluating the efficacy of PDAC chemoradiotherapy and neoadjuvant chemotherapy [17, 18].

The components of the TME are complex, and CAFs have various subtypes whose functions remain unclear. Relying solely on the analysis of a proportion of CAFs in the TME for prognostic prediction using pathological methods may offer a limited perspective for patients with PDAC. Additionally, interobserver disagreement may occur in assessing the boundary of the lesion during image review, particularly when the attenuation or contours of certain PDAC are indistinguishable from pancreatic morphological variations [19]. Therefore, this study aimed to assess the ECM of pancreatic cancer using the fECV derived from enhanced CT images and integrating fECV with α‐SMA‐positive CAFs and FAP‐positive CAFs to investigate their relation to clinicopathological characteristics and prognosis in patients with PDAC.

## 2 Materials and Methods

### 2.1 Patient Population

**FIGURE 1:**
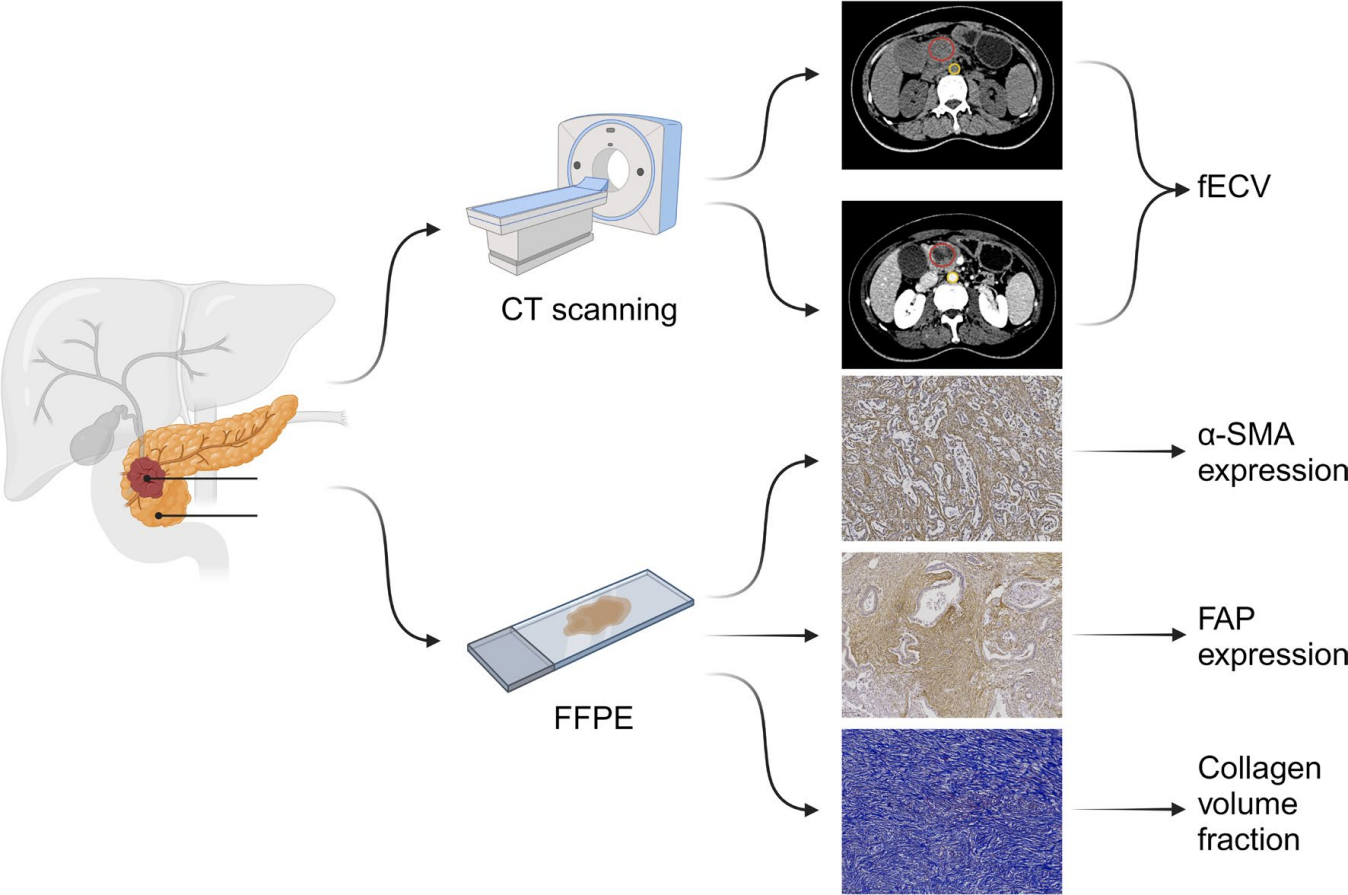
A cohort of 124 patients was enrolled based on inclusion and exclusion criteria. All patients underwent preoperative multiple phase CT scans, and postoperative paraffin‐embedded tissue specimens were collected for staining analysis. The fECV was calculated using absolute enhancement of attenuation values between equilibrium and non‐enhanced scans of PDAC and aorta according to Formula (1). The staining results were quantitatively analyzed using ImageJ software to calculate the collagen volume fraction and the expression levels of α‐SMA and FAP.

Patients who had undergone surgical intervention for PDAC at the Sun Yat‐sen Memorial Hospital, Sun Yat‐sen University between 2014 and 2021 were included. This study received approval from the institutional review committee, and the requirement for informed consent was waived. Ultimately, 124 patients with postoperative chemotherapy and were included in the final study cohort. The inclusion criteria, data collection, treatment regimens, and efficacy assessment of treatment are provided in the Supporting Information. The flowchart summarizing the experimental design and data collection methods is shown in Figure 1.

### 2.2 Immunohistochemistry (IHC), Multiplex Immunohistochemistry (mIHC), and Masson's Trichrome Staining

The IHC and mIHC staining procedures were the same as the protocols outlined in our previous study [20]. Detailed staining protocols, specific reagents used in the staining process, and methods of staining analysis are provided in the Supporting Information. Pathologists involved in the analysis are unaware of the patient's clinical information.

### 2.3 CT Imaging Technology

All the patients in the study cohort underwent abdominal contrast‐enhanced CT using a 64‐row multidetector CT scanner (Discovery CT750hd; GE Healthcare, Waukesha, Wisconsin).

Two surgeons, with 8 and 6 years of experience respectively, with professional imaging training evaluated the CT images independently. Subsequently, a board‐certified radiologist with 12 years of experience reviewed the results manually. In cases of discrepancies, another board‐certified radiologist with 21 years of experience was involved to achieve a consensus. All surgeons and radiologists involved in the analysis are unaware of the patient's clinical information.

**FIGURE 2:**
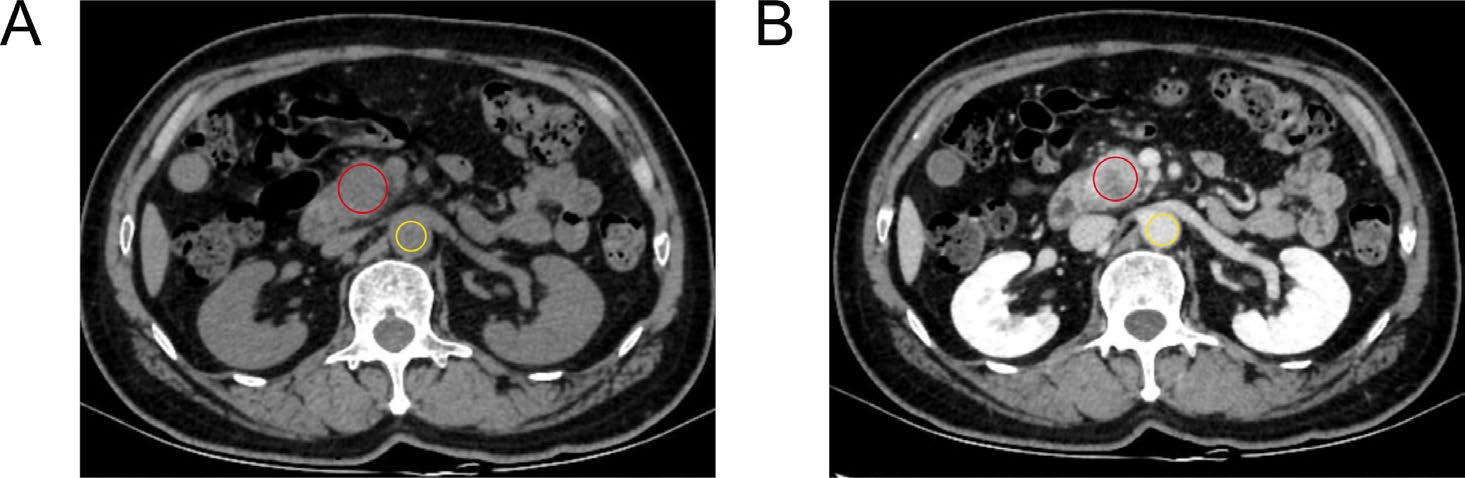
In the non‐enhanced axial scans (A) and the equilibrium axial scans (B) of CT image, ROI selection is performed (the red ellipse represents the ROI of the PDAC lesion, and the yellow ellipse represents the ROI of the abdominal aorta). (A) In the non‐enhanced image, attenuation values of the PDAC lesion and the abdominal aorta are 37 HU and 47 HU, respectively. (B) In the equilibrium phase image, attenuation values of the PDAC lesion and the abdominal aorta are 92 HU and 172 HU, respectively. This patient's HCT is 0.422, ΔHU^tumor^ is 55, ΔHU^aorta^ is 125. According to Formula (1), the patient's fECV is calculated to be 25.43.

The process of acquiring fECV involved delineating regions of interest (ROI) that were oval within the largest cross‐section of the PDAC lesion on both non‐enhanced axial scans and equilibrium phase axial scans. The ROI of pancreatic cancer lesions should include all PDAC lesions as much as possible, and the abdominal aorta was selected as the ROI for the same images (Figure 2). Measurements were performed at three consecutive levels, and the average value was determined.

Based on the attenuation values and hematocrit (HCT) of peripheral blood, Formula (1) was used to calculate fECV [15]. For more CT imaging technical details, please refer to the Supporting Information.

(1) $$\text{fECV}\;\left(\%\right)=\left(1-\mathrm{HCT}\right)\times \left({\mathrm{\Delta HU}}^{\text{tumor}}/{\mathrm{\Delta HU}}^{\text{aorta}}\right)\times 100$$

### 2.4 Statistical Analysis

Clinicopathological characteristics were examined using the chi‐square test or Fisher's exact test. Differences between groups were analyzed using either the Kruskal–Wallis H test or the Mann–Whitney *U* test. Pearson or Spearman correlation analyses were conducted to assess the correlation between fECV and collagen volume fraction. Interobserver agreement for fECV measurements on contrast‐enhanced CT was assessed using the intraclass correlation coefficient (ICC) with absolute agreement under a two‐way random‐effects model. The Kaplan–Meier (K–M) method was employed to plot the postoperative OS and disease‐free survival (DFS) curves. The log‐rank test is applied to compare survival disparities between the groups. A Cox proportional hazards regression model was utilized to develop a predictive model. A nomogram was constructed for prognostic prediction. The prediction accuracy of the model was assessed using the receiver operating characteristic (ROC) curves. Additionally, tenfold cross‐validation was performed to validate the model. Calibration and decision curve analysis (DCA) curves were plotted to assess the performance of the predictive model. All data analyses were performed using SPSS 26.0 (IBM Corp., Armonk, NY, USA) and R software 4.3.2 (R Core Team, Vienna, Austria). For more statistical analysis details, please refer to the Supporting Information.

## 3 Results

### 3.1 Clinicopathological Characteristics of PDAC Research Cohorts

**FIGURE 3:**
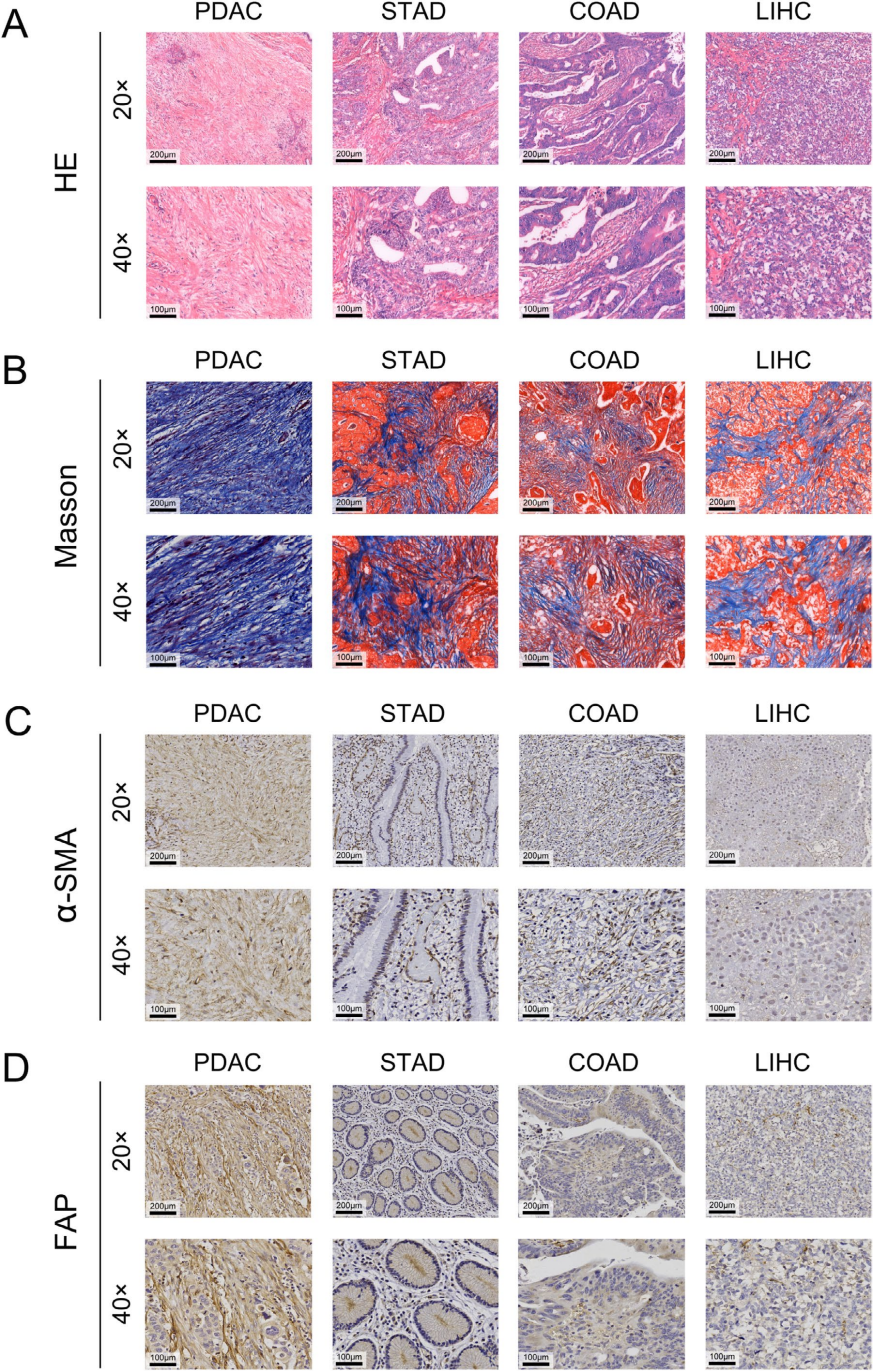
In gastrointestinal malignancies, PDAC exhibits a higher degree of fibrosis and higher levels of α‐SMA and FAP expression compared to stomach adenocarcinoma (STAD), colon adenocarcinoma (COAD), and liver hepatocellular carcinoma (LIHC). (A, B) Representative images of HE staining and Masson's trichrome staining of PDAC, STAD, COAD, and LIHC at 20× and 40× magnification. HE staining of PDAC shows the most extracellular matrix and collagen fibers deposition. (C,D) Representative images of α‐SMA and FAP IHC staining of PDAC, STAD, COAD, and LIHC at 20× and 40× magnification. IHC staining results show that PDAC has the highest level of α‐SMA and FAP expression.

**FIGURE 4:**
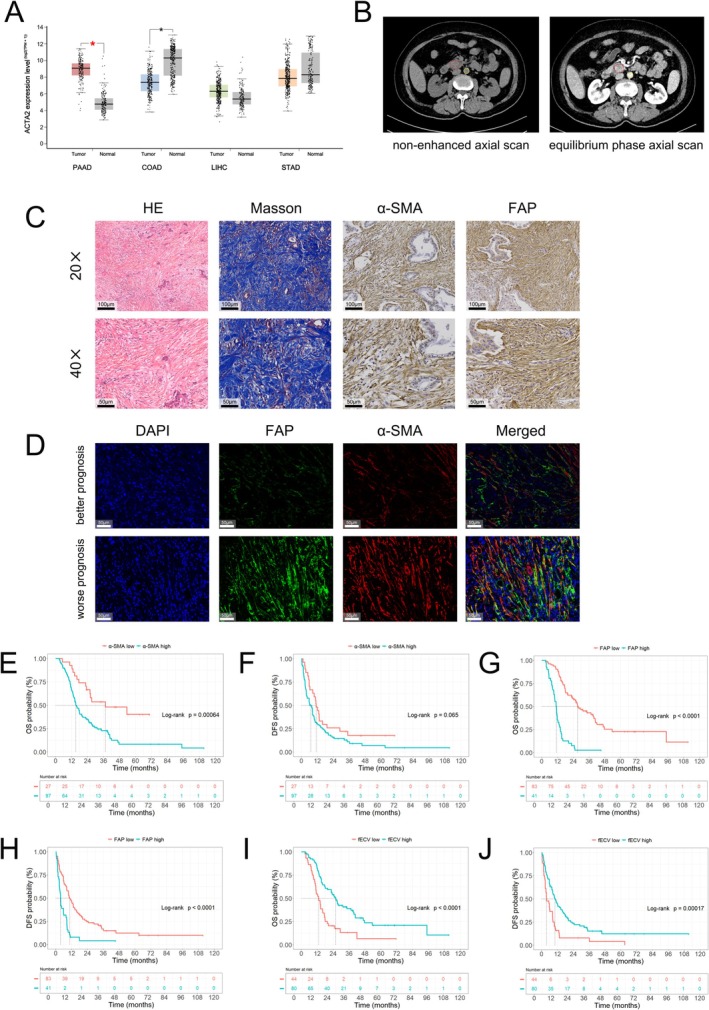
High‐specificity expression of α‐SMA, elevated expression of FAP and a reduced level of fECV are associated with poor prognosis. (A) Expression of ACTA2 in tumor (PAAD, COAD, LIHC and STAD) and normal tissue. TCGA data shows that PAAD has higher ACTA2 expression, which encodes α‐SMA, compared to normal tissue. (B) Representative image showing the ROI selection for fECV measurement. (C) Representative images of HE, Masson, α‐SMA and FAP IHC staining of PDAC at 20× and 40× magnification. (D) Representative images of α‐SMA and FAP mIHC staining of PDAC at 40× magnification. The nucleus, α‐SMA, and FAP are labeled with DAPI (blue), red, and green, respectively. The patient in the lower panel exhibits higher levels of FAP and α‐SMA expression and has a poorer prognosis compared to the patient in the upper panel (OS: 16.1 months vs. 45.1 months). (E, F) K–M survival curves for OS and DFS in patients with high and low α‐SMA expression. (G, H) K–M survival curves for OS and DFS in patients with high and low FAP expression. (I, J) K–M survival curves of OS and DFS for patients with high and low fECV.

The clinicopathological characteristics of the 124 patients with PDAC are summarized in Table S1. In addition, all patients with distant metastasis have liver oligometastasis and underwent simultaneous resection of the metastatic lesion. As shown in Figure 3, compared with other types of cancer, PDAC α‐SMA and FAP exhibit more severe fibrosis, with higher expression levels of α‐SMA and FAP. Additionally, data from the Genomic Data Commons program (GDC, https://portal.gdc.cancer.gov/ [21]) indicate that the ACTA2, which encodes α‐SMA, is specifically upregulated in pancreatic adenocarcinoma (PAAD) (Figure 4A).

The immunohistochemical staining results and representative images of ROI delineation are shown in Figure 4B–D. The fECV was calculated, and an intragroup correlation analysis was performed on fECV values measured independently by two physicians. ICC for fECV demonstrated a strong interobserver agreement (ICC = 0.861, 95% confidence interval: 0.802–0.903, *p* < 0.001). The cutoff values for each indicator were determined using X‐tile software (Table S2).

### 3.2 Relationship Between α‐SMA, FAP Expression, fECV, and Clinicopathological Characteristics

The association between α‐SMA and FAP expression and various clinicopathological parameters is summarized in Table 1. The α‐SMA expression (*p* = 0.049) was elevated in a substantial proportion of men. Additionally, high α‐SMA expression was connected with lesions predominantly localized in the head or neck of the pancreas (*p* = 0.010) and lower tumor differentiation (*p* = 0.013). High expression of FAP was related to higher levels of the tumor marker CA19‐9 (*p* = 0.012). Considering the association between poor prognosis and the elevated expression of α‐SMA and FAP, patients exhibiting co‐high expression of α‐SMA and FAP were analyzed as a distinct subgroup. Notably, co‐high expression of α‐SMA and FAP was linked to higher CA19‐9 levels (*p* = 0.016) and elevated CA125 (*p* = 0.042).

**TABLE 1:** The correlation between α‐SMA and FAP expression status and clinicopathological characteristics in PDAC.

Characteristic	Cases (*n*)	α‐SMA expression			FAP expression			α‐SMA + FAP co‐high expression		
		High	Low	*p*	High	Low	*p*	Present	Absent	*p*
Age (years)				0.664			0.455			0.433
≤ 60	62	47	15		18	44		16	46	
> 60	62	50	12		23	39		21	41	
Gender				**0.049**			0.187			0.328
Male	68	58	10		26	42		23	45	
Female	56	39	17		15	41		14	42	
CA 19‐9 (U/mL)				0.827			**0.012**			**0.016**
≤ 500	72	57	15		17	55		15	57	
> 500	52	40	12		24	28		22	30	
CEA (ng/mL)				0.501			0.432			0.314
≤ 5	79	60	19		24	55		21	58	
> 5	45	37	8		17	28		16	29	
CA125 (U/mL)				1.000			0.124			**0.042**
≤ 35	93	73	20		27	66		23	70	
> 35	31	24	7		14	17		14	17	
Bile duct dilatation				0.280			0.705			0.436
Yes	63	52	11		22	41		21	42	
No	61	45	16		19	42		16	45	
Pancreatic duct dilatation				0.177			0.322			0.415
Yes	79	65	14		29	50		26	53	
No	45	32	13		12	33		11	34	
Location				**0.010**			0.824			0.493
Head/neck	94	79	15		32	62		30	64	
Body/tail	30	18	12		9	21		7	23	
AJCC prognostic stage				0.714			0.338			0.108
I	28	21	7		6	22		4	24	
II	48	40	8		17	31		16	32	
III	31	23	8		10	21		9	22	
IV	17	13	4		8	9		8	9	
T stage				0.935			0.233			0.069
T1	9	8	1		2	7		2	7	
T2	64	49	15		18	46		15	49	
T3	39	30	9		18	21		18	21	
T4	12	10	2		3	9		2	10	
N stage				0.307			0.314			0.170
N0	50	36	14		16	34		14	36	
N1	47	40	7		13	34		11	36	
N2	27	21	6		12	15		12	15	
M stage				1.000			0.226			0.151
M0	107	84	23		33	74		29	78	
M1	17	13	4		8	9		8	9	
Histological differentiation				**0.013**			0.333			0.166
Well	9	7	2		2	7		1	8	
Moderate	69	48	21		20	49		18	51	
Poor	46	42	4		19	27		18	28	
Vascular invasion				0.562			1.000			0.607
Yes	21	18	3		7	14		5	16	
No	103	79	24		34	69		32	71	
Perineural invasion				1.000			1.000			1.000
Yes	108	84	24		36	72		33	75	
No	16	13	3		5	11		4	12	

*Note:* Bold values indicate statistically significance at *p* < 0.05.

Based on fECV levels, the patient cohort was categorized, and the grouping outcomes are detailed in Table 2. Lower fECV levels were associated with advanced T stage (*p* = 0.025), higher levels of CA19‐9 (*p* = 0.014), and elevated CEA levels (*p* = 0.031). Meanwhile, fECV correlated with upregulated FAP expression (*p* = 0.028), whereas no such correlation was observed with high α‐SMA expression (*p* = 0.199). Moreover, a lower fECV was correlated with a high expression of FAP (*p* = 0.045). The fECV was markedly lower in the α‐SMA^high^ + FAP^high^ group compared to the α‐SMA^low^ + FAP^low^ group (*p* < 0.001). Additionally, fECV, SMA, and FAP were not associated with N stage or M stage.

**TABLE 2:** The correlation between fECV and clinicopathological characteristics in PDAC.

Clinical parameters	Cases (*n*)	fECV			α‐SMA high expression + fECV low		
		High	Low	*p*	Present	Absent	*p*
Age (years)				0.590			0.553
≤ 60	62	42	20		16	46	
> 60	62	38	24		20	42	
Gender				1.000			1.000
Male	68	44	24		20	48	
Female	56	36	20		16	40	
CA 19‐9 (U/mL)				**0.014**			0.071
≤ 500	72	53	19		16	56	
> 500	52	27	25		20	32	
CEA (ng/mL)				**0.031**			**0.023**
≤ 5	79	62	22		17	62	
> 5	45	18	22		19	26	
CA125 (U/mL)				0.395			0.654
≤ 35	93	45	31		26	67	
> 35	31	35	13		10	21	
Bile duct dilatation				0.133			0.430
Yes	63	53	18		16	47	
No	61	27	26		20	41	
Pancreatic duct dilatation				0.442			0.837
Yes	79	53	26		22	57	
No	45	27	18		14	31	
Location				0.079			0.493
Head/neck	94	65	29		26	68	
Body/tail	30	15	15		10	20	
AJCC prognostic stage				0.190			0.194
I	28	22	6		4	24	
II	48	31	17		15	33	
III	31	19	12		10	21	
IV	17	8	9		7	10	
T stage				**0.025**			**0.037**
T1	9	8	1		0	9	
T2	64	45	19		16	48	
T3	39	18	21		17	22	
T4	12	9	3		3	9	
N stage				0.581			0.354
N0	50	35	15		11	39	
N1	47	29	18		16	31	
N2	27	16	11		9	18	
M stage				0.171			0.257
M0	107	72	35		29	78	
M1	17	8	9		7	10	
Histological differentiation				0.769			0.704
Well	9	5	4		3	6	
Moderate	69	46	23		18	51	
Poor	46	29	17		15	31	
Vascular invasion				0.219			0.185
Yes	21	11	10		9	12	
No	103	69	34		27	76	
Perineural invasion				0.577			1.000
Yes	108	71	37		30	78	
No	16	9	7		6	10	

*Note:* Bold values indicate statistically significant at *p* < 0.05.

### 3.3 High Expression of α‐SMA and FAP and Low fECV Are Predictors of Worse Prognosis

**FIGURE 5:**
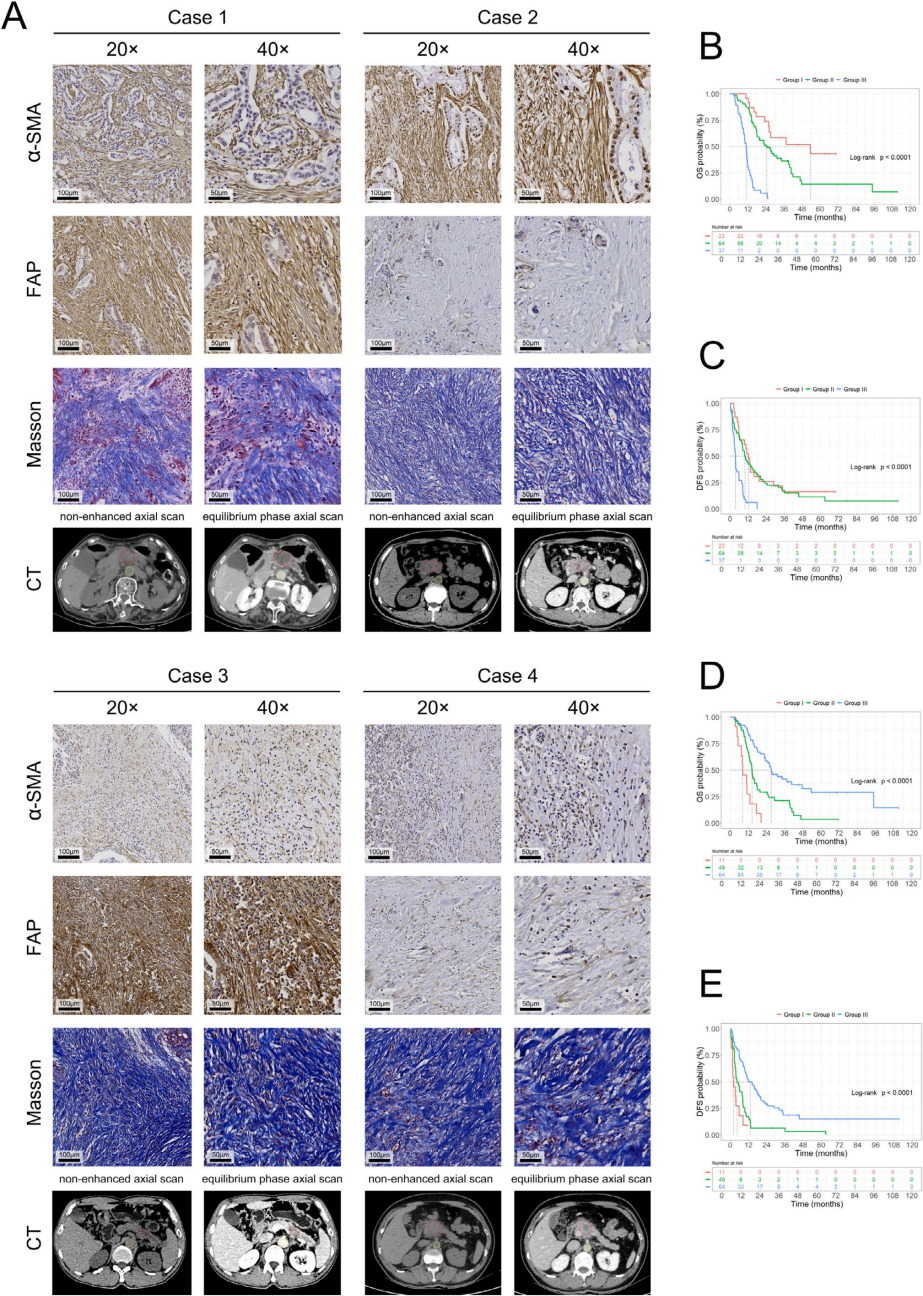
Co‐expression high expression of α‐SMA and FAP and high expression of α‐SMA combined with low fECV are associated with poor prognosis. (A) Representative images of ROI selection for fECV measurement and IHC and Masson staining of PDAC at 20× and 40× magnification in four different cases based on the expression status of α‐SMA and FAP. (B, C) K–M survival curves for postoperative OS and PFS of PDAC patients according to co‐expression status of α‐SMA and FAP (Note: Group I: α‐SMA^low^ + FAP^low^; Group II: α‐SMA^high^ + FAP^low^ and α‐SMA^low^ + FAP^high^; Group III: α‐SMA^high^ + FAP^high^). (D, E) K–M survival curves for postoperative OS and PFS of PDAC patients according to expression status of α‐SMA and fECV (Note: Group I: α‐SMA^high^ + fECV^low^; Group II: α‐SMA^high^ + fECV^high^ and α‐SMA^low^ + fECV^low^; Group III: α‐SMA^low^ + fECV^high^).

Survival analysis based on α‐SMA and FAP expression was performed (Figure 4E–H). The subset with elevated co‐expression of α‐SMA and FAP exhibited the shortest mOS and mDFS (mOS: 10.9 months and mDFS: 3.6 months). The results revealed a notable association between elevated α‐SMA expression and reduced OS (*p* < 0.001), whereas α‐SMA expression was not statistically correlated with DFS (*p* = 0.06). Similarly, the cohort with elevated FAP expression demonstrated significantly reduced OS (*p* < 0.001) and DFS (*p* < 0.001). By analyzing the co‐expression of α‐SMA and FAP (Figure 5B,C), we found that the OS in Group III was significantly shorter than that of both Group II (*p* < 0.001) and Group I (*p* < 0.001). Additionally, OS was higher in Group I than in Group II (*p* = 0.026). The DFS in Group III was also notably shorter than in Group II (*p* < 0.001) and Group I (*p* < 0.001). In summary, high co‐expression of α‐SMA and FAP is associated with a poor prognosis.

Compared to the low fECV group, mOS was longer in the high fECV group (23.7 vs. 13.0 months, *p* < 0.001). Additionally, the high fECV group exhibited longer mDFS (9.9 vs. 3.8 months, *p* < 0.001). The K–M curves for OS in both groups are illustrated in Figure 4I,J, indicating that patients with higher fECV exhibit longer OS (*p* < 0.001) and longer DFS (*p* < 0.001).

Due to the relationship between high α‐SMA expression and low fECV with poor prognosis, patients were categorized into three groups for survival analysis (Figure 5D,E). Patients in Group I (α‐SMA^high^ + fECV^low^) had shorter OS than those in Group II (*p* = 0.003) and Group III (*p* < 0.001), whereas OS was significantly longer among patients in Group III than in Group II (*p* < 0.001) (Figure 5D). No significant difference in DFS was observed between Groups I and II (*p* = 0.104); however, DFS was significantly longer in Group III than in Group II (*p* < 0.001) and Group I (*p* < 0.001). The combination of high α‐SMA expression and low fECV is associated with a poor prognosis.

### 3.4 fECV Combined With Expression of α‐SMA and FAP for Predicting Pancreatic Cancer Survival

Univariate Cox regression analysis (Table 3) revealed that female sex (*p* = 0.014), elevated levels of CA19‐9 (*p* = 0.002), N2 lymph node metastasis (*p* = 0.002), distant metastasis (*p* = 0.005), histological undifferentiation (*p* = 0.038), high expression of α‐SMA (*p* = 0.001), high expression of FAP (*p* < 0.001), and lower fECV (*p* < 0.001) were associated with poorer prognosis. Additionally, the univariate Cox regression results indicated that patients receiving GnP (Gemcitabine + Nab‐Paclitaxel) chemotherapy had a better OS than those receiving FOLFIRINOX chemotherapy (*p* = 0.004).

**TABLE 3:** Univariate and multivariate regression analysis of prognostic factors correlated with postoperative OS.

Clinical parameters	Univariate analysis		Multivariate analysis		Backward elimination	
	Hazard ratio (95% CI)	*p*	Hazard ratio (95% CI)	*p*	Hazard ratio (95% CI)	*p*
Age (years)						
≤ 60	1 (ref)	—	—	—	—	—
> 60	1.37 (0.91–2.06)	0.137	—	—	—	—
Gender						
Male	1 (ref)	—	1 (ref)	—	—	—
Female	1.69 (1.11–2.57)	**0.014**	1.19 (0.73–1.95)	0.488	—	—
Hypertension						
No	1 (ref)	—	—	—	—	—
Yes	0.69 (0.41–1.15)	0.153	—	—	—	—
Diabetes						
No	1 (ref)	—	—	—	—	—
Yes	1.30 (0.80–2.10)	0.291	—	—	—	—
CA 19‐9 (U/mL)						
≤ 500	1 (ref)	—	1 (ref)	—	—	—
> 500	1.92 (1.26–2.93)	**0.002**	1.50 (0.94–2.40)	0.089	—	—
CEA (ng/mL)						
≤ 5	1 (ref)	—	—	—	—	—
> 5	1.36 (0.90–2.07)	0.148	—	—	—	—
CA125 (U/mL)						
≤ 35	1 (ref)	—	—	—	—	—
> 35	1.30 (0.82–2.07)	0.259	—	—	—	—
fECV						
Low	1 (ref)	—	1 (ref)	—	1 (ref)	—
High	0.42 (0.28–0.65)	**< 0.001**	0.57 (0.36–0.93)	**0.023**	0.58 (0.37–0.90)	**0.015**
Bile duct dilatation						
No	1 (ref)	—	—	—	—	—
Yes	1.04 (0.69–1.56)	0.865	—	—	—	—
Pancreatic duct dilatation						
No	1 (ref)	—	—	—	—	—
Yes	1.38 (0.90–2.12)	0.144	—	—	—	—
Location						
Head/neck	1 (ref)	—	—	—	—	—
Body/tail	0.66 (0.39–1.10)	0.110	—	—	—	—
T stage						
T1	1 (ref)	—	—	—	—	—
T2	0.68 (0.32–1.45)	0.323	—	—	—	—
T3	0.83 (0.38–1.83)	0.645	—	—	—	—
T4	0.76 (0.29–1.98)	0.572	—	—	—	—
N stage						
N0	1 (ref)	—	1 (ref)	—	1 (ref)	—
N1	1.32 (0.83–2.11)	0.238	1.36 (0.83–2.22)	0.217	1.40 (0.88–2.25)	0.159
N2	1.72 (1.00–2.95)	**0.048**	1.58 (0.86–2.89)	0.140	1.88 (1.08–3.27)	**0.025**
M stage						
M0	1 (ref)	—	1 (ref)	—	1 (ref)	—
M1	2.19 (1.26–3.79)	**0.005**	1.88 (1.03–3.43)	**0.041**	1.87 (1.06–3.31)	**0.031**
Histological differentiation						
Poor	1 (ref)	—	1 (ref)		—	—
Moderate	0.63 (0.41–0.97)	**0.038**	0.74 (0.46–1.21)	0.228	—	—
Well	0.61 (0.27–1.38)	0.238	0.88 (0.37–2.10)	0.769	—	—
Vascular invasion						
Yes	1 (ref)	—	—	—	—	—
No	1.31 (0.78–2.21)	0.303	—	—	—	—
Perineural invasion						
Yes	1 (ref)	—	—	—	—	—
No	1.13 (0.61–2.08)	0.691	—	—	—	—
FAP expression						
Low	1 (ref)	—	1 (ref)	—	1 (ref)	—
High	5.16 (3.29–8.11)	**< 0.001**	3.88 (2.29–6.55)	**< 0.001**	4.43 (2.72–7.19)	**< 0.001**
α‐SMA expression						
Low	1 (ref)	—	1 (ref)	—	1 (ref)	—
High	2.63 (1.48–4.67)	**0.001**	1.76 (0.93–3.35)	0.083	2.34 (1.30–4.21)	**0.005**
Chemotherapy						
FOLFIRINOX	1 (ref)	—	1 (ref)	—	—	—
GS	0.78 (0.43–1.39)	0.397	0.85 (0.46–1.57)	0.606	—	—
AG	0.39 (0.20–0.75)	**0.005**	0.54 (0.25–1.14)	0.108	—	—
Gemcitabine alone	0.73 (0.34–1.54)	0.717	1.16 (0.51–2.63)	0.717	—	—
S‐1 alone	0.89 (0.46–1.72)	0.720	0.90 (0.43–1.86)	0.768	—	—

*Note:* Bold values indicate statistically significance at *p* < 0.05.

**FIGURE 6:**
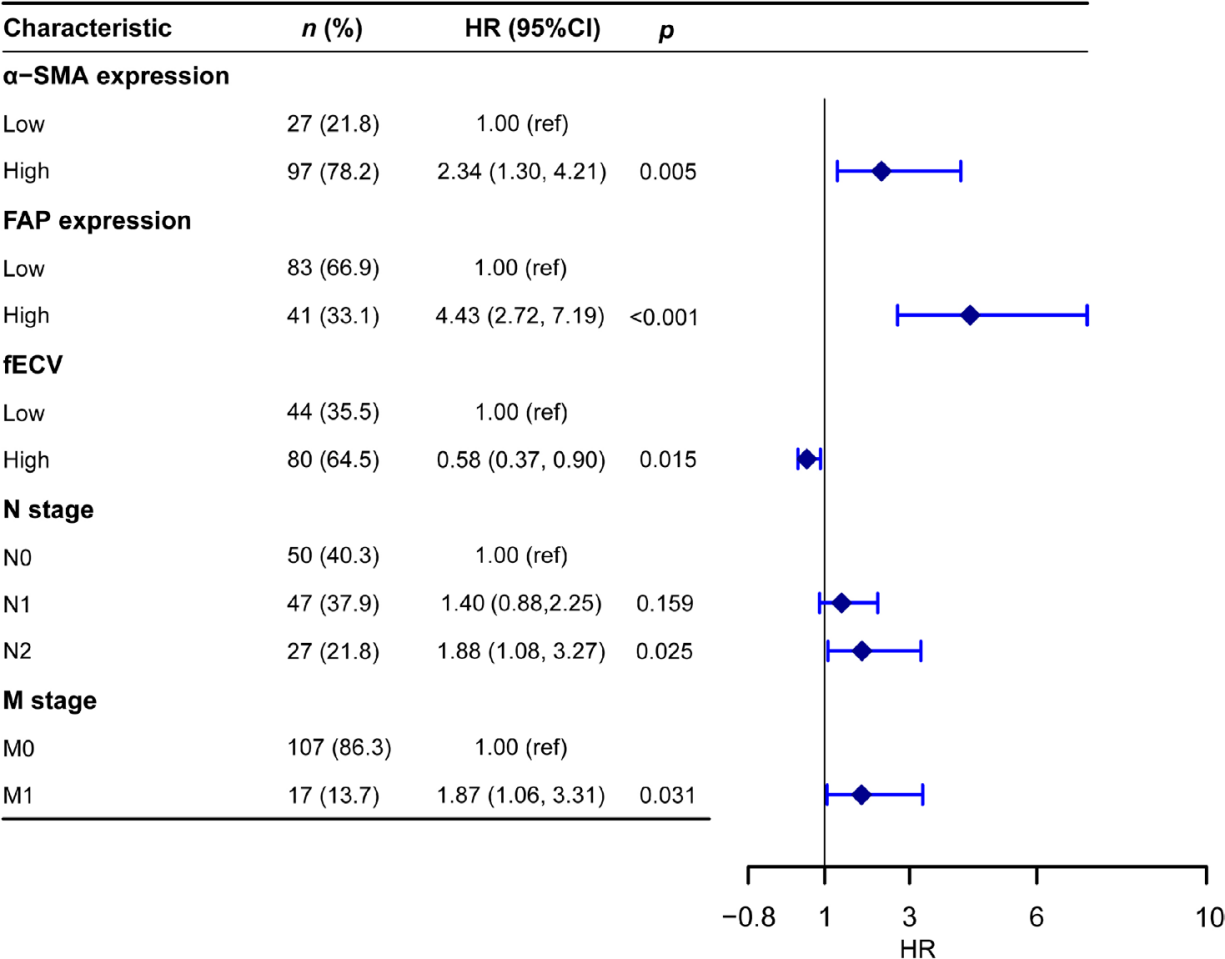
Forest plot based on the results of multivariable Cox regression analysis.

As illustrated in the forest plot (Figure 6), the results of multivariable Cox regression analysis revealed that lymph node metastasis (*p* = 0.025), distant liver metastasis (*p* = 0.031), high expression of α‐SMA (*p* = 0.005), high expression of FAP (*p* < 0.001), and lower fECV (*p* = 0.015) were independent risk factors for patients undergoing postoperative chemotherapy for PDAC.

**FIGURE 7:**
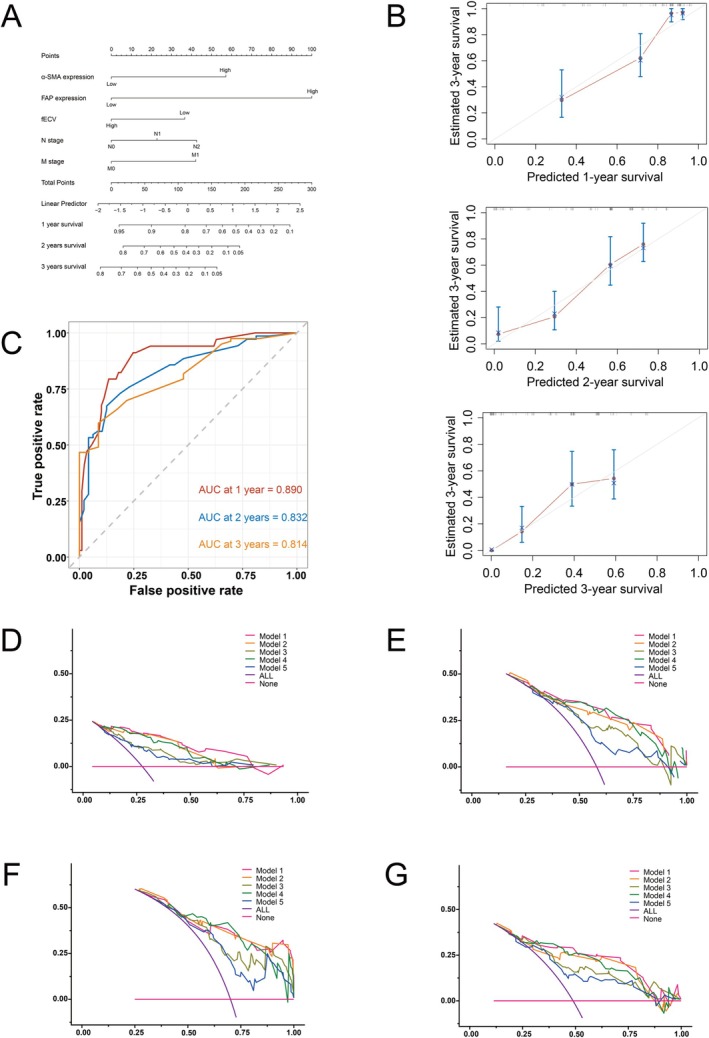
The predictive model established by our research cohort demonstrates favorable prognostic efficacy for OS in PDAC patients. (A) The nomogram for predicting 1‐, 2‐, and 3‐year OS in PDAC patients. (B) The calibration curves of the nomogram in the study cohort. (C) ROC curves of the study cohort for predicting 1‐, 2‐, and 3‐year OS. The AUC of the ROC curves for predicting 1‐, 2‐, and 3‐year survival rates was 0.890, 0.832, and 0.814, respectively. (D–G), Decision curve analysis of all models for predicting 1‐year, 2‐year, 3‐year, and median OS. AUDC of each model is listed in Table 4.

A nomogram was developed to estimate patient survival probabilities, informed by the results of the multivariate Cox regression analysis (Figure 7A). The average area under the curve (AUC) of ten‐fold cross‐validation was 0.847, suggesting a robust generalization performance of the model. The ROC curves derived from the nomogram are shown in Figure 7C. The AUCs of the ROC curves for predicting 1‐, 2‐, and 3‐year survival rates were 0.890, 0.832, and 0.814, respectively. Furthermore, calibration curves plotted to validate the nomogram (Figure 7B) indicated good consistency between the predicted and observed values. Patients were stratified into low‐ and high‐risk groups based on nomogram scores for prognosis analysis (Figure S1B), which revealed significantly longer survival periods in the low‐risk group than in the high‐risk group (*p* < 0.001).

Multiple Cox proportional hazards regression models were established, as shown in Table S3. Each model was subjected to tenfold cross‐validation. Additionally, DCA was employed to assess the accuracy of the models.

The DCA curves for each model are presented in Figure 7D–G, and the average AUC of tenfold cross‐validation and the area under the decision curve (AUDC) for each model are shown in Table 4. Among them, Model 1, which is based on clinicopathological characteristics + expression of α‐SMA and FAP + fECV, has the highest average AUC and the highest AUDC for the assessment of 1‐, 2‐, and 3‐year survival periods across all models, indicating the best generalization ability and the highest accuracy in evaluating the 1‐ to 3‐year survival periods of patients with PDAC.

**TABLE 4:** Average AUC of tenfold cross‐validation and the AUDC for each model.

Model	Mean AUC of *k* fold CV	Area under DCA curve			
		1‐year OS	2‐year OS	3‐year OS	Median OS
Model 1	0.847	0.104	0.245	0.301	0.206
Model 2	0.783	0.084	0.228	0.294	0.187
Model 3	0.748	0.060	0.191	0.258	0.160
Model 4	0.776	0.073	0.181	0.200	0.156
Model 5	0.747	0.047	0.151	0.213	0.127

### 3.5 Predictive Value of fECV for the Efficacy of Different Chemotherapy Regimens

Owing to the apparent influence of chemotherapy regimens on prognosis in univariate analysis, K–M curves (Figure S1A) were plotted for analysis. The results showed that patients treated with the GnP regimen had longer OS than those treated with the FOLFIRINOX regimen (*p* = 0.001), GS regimen (*p* = 0.011), and S‐1 monotherapy (*p* = 0.001). To further explore the effect of fibrotic extracellular volume (fECV) on survival in chemotherapy patients, the study cohort was expanded to include 25 additional PDAC patients who received combination postoperative chemotherapy. The mOS for the GnP regimen group was 25.2 months, which was significantly longer than those in the FOLFIRINOX regimen group (15.2 months, *p* = 0.011) and the GS regimen group (15.2 months, *p* < 0.001). Survival analysis of OS with different combination chemotherapy regimens based on the fECV high‐low stratification was conducted. In the high fECV group (Figure S2C), no statistically significant difference in OS was observed between patients receiving the GnP chemotherapy regimen and those receiving the GS regimen (*p* = 0.091) or FOLFIRINOX regimen (*p* = 0.394). In the low fECV group (Figure S2D), patients treated with the AG regimen had a longer OS than those treated with the GS regimen (*p* = 0.041) and the FOLFIRINOX regimen (*p* = 0.016).

## 4 Discussion

The TME of PDAC from both radiological and pathological perspectives, using fECV and the CAFs markers α‐SMA and FAP, was evaluated in this study. A lower fECV was associated with higher CA19‐9 levels. In patients with lower fECV, CA19‐9 levels tend to increase gradually from the beginning to the end of chemotherapy [18], indicating a poorer response to chemotherapy. Survival analysis revealed that lower fECV levels are associated with worse OS and DFS in patients undergoing postoperative chemotherapy for PDAC, suggesting that fECV is a potential prognostic indicator.

The iodine concentration within tumor lesions on contrast‐enhanced CT scans is influenced not only by the blood flow status of the TME but also by technical factors and the hemodynamic status of the patient [22]. The fECV is a reliable and stable quantitative parameter that is unaffected by various technical and physiological confounders [22]. Based on the correlation analysis between collagen volume fraction and fECV, our study confirmed a weak positive linear correlation between fECV and extracellular collagen in pancreatic cancer (correlation coefficient, *R* = 0.281, *p* = 0.002). This indicates that the fECV can reflect the ECM of pancreatic cancer lesions to a certain extent.

Current research suggests that a dense ECM in pancreatic cancer contributes to a poor prognosis in patients [23]. Numerous studies have indicated that the increased tissue tension and stiffness resulting from a highly fibrotic ECM enhance tumor cell invasiveness and contribute to cancer spread [24, 25, 26, 27]. Recent research has shown that CXCL3‐induced myCAFs express high levels of collagen III on their surface. These myCAFs interact with PDAC cells via integrins, facilitating the formation of metastatic myCAFs–PDAC clusters, which carry PDAC cells to distant sites [28]. Additionally, myCAFs may play a role in the formation of metastatic niches and the growth of metastases in distant organs such as the liver [28]. This suggests that the TME of distant metastatic lesions may resemble that of primary PDAC tumors. Genetic studies have revealed significant similarities between primary and metastatic lesions, with the stromal components surrounding cancer cells being relatively similar in both primary and metastatic PDAC lesions [29].

Previous research has demonstrated that evaluating the fECV of the primary lesion has prognostic value for stage IV PDAC patients undergoing chemotherapy [16]. As fECV reflects the overall characteristics of the PDAC microenvironment and partial biological characteristics of tumors, assessing the fECV of the primary lesion might predict the efficacy of chemotherapy in inhibiting potential micrometastases with similar microenvironmental features. Our study findings were consistent with those of previous studies [17, 18, 30] indicating that a low fECV is associated with a poor prognosis because fECV is affected by the proportion of ECM and vascular distribution, suggesting that fECV may partially predict the efficacy of chemotherapy in inhibiting micrometastases in these patients. A highly fibrotic TME leads to reduced vascular proliferation, resulting in inadequate blood perfusion with iodine contrast agents. Moreover, the densely packed ECM not only impedes the diffusion of small‐molecule drugs but may also hinder the distribution of iodine contrast agents, causing a decrease in fECV. This mechanism is similar to the effects of the microenvironment on chemotherapeutic drugs. Therefore, in PDAC, the fECV may be correlated to some extent with the intratumoral concentration of chemotherapy drugs.

The invasiveness of PDAC is mainly attributed to the increased stromal fibrosis in PDAC [12]. MyCAFs increase the secretion of carcinogenic collagen type I α1 (Col1α1) through the TGF‐β/SMAD signaling pathway, contributing to the formation of the ECM [31]. This also indicates the impact of α‐SMA expression and matrix density on prognosis. Our findings elucidated that high expression of α‐SMA was correlated with poor prognosis, aligning with those of previous studies [5, 9, 32]. Moreover, high expression of α‐SMA was linked to poor tumor differentiation. In the α‐SMA^high^ group, more than 40% of patients had poorly differentiated PDAC, in contrast to only 15% in the α‐SMA^low^ group, which highlights that high expression of α‐SMA is a marker of stronger tumor invasion and a worse prognosis. However, α‐SMA expression did not demonstrate a significant impact on DFS (*p* = 0.065), which may be attributed to the relatively small number of patients with low α‐SMA expression (*n* = 27), warranting further investigation.

Previously, studies have suggested that high FAP expression of PDAC is associated with increased stromal fibrosis and poor prognosis [10, 11]. In this study, FAP expression was associated with higher levels of clinical tumor markers, indicating a poorer prognosis. Moreover, the results of the survival analysis demonstrated that high FAP expression predicts worse OS and DFS.

Currently, chemotherapy is the primary treatment for relieving PDAC symptoms and prolonging survival. Fibrotic changes in pancreatic cancer lesions lead to vascular dysfunction, resulting in alterations in interstitial fluid pressure, ultimately manifesting as poor drug penetration within the TME [33]. Albumin‐bound paclitaxel is a combination of traditional paclitaxel and albumin nanoparticles that bind to cysteine‐rich acidic secretory proteins in the tumor and matrix, thereby enhancing the selective delivery of paclitaxel in PDAC [34]. Patients with locally advanced PDAC with a high proportion of tumor stroma benefit from the GnP regimen but not from other gemcitabine‐based regimens [35]. Endoscopic ultrasound elastography performed in patients receiving two cycles of the GnP regimen before surgical resection for advanced pancreatic cancer revealed a reduction in tumor stiffness [36], and the histopathological findings demonstrated a reduction in α‐SMA‐positive CAFs [37] and collagen fibers [36], which underscore that the GnP regimen can significantly alter the tumor stroma and lead to tumor “softening” [36]. In this study, the GnP regimen resulted in the highest mOS. Specifically, in the low fECV group, where ECM was more abundant, patients receiving the GnP regimen exhibited significantly improved OS than those receiving the FOLFIRINOX and GS regimens. This could be attributed to the considerable advantage of albumin‐bound paclitaxel in targeting the ECM in individuals with a more abundant ECM. The GnP regimen is effective in reducing the ECM [37]. A study comparing the efficacy of the GnP regimen and sequential chemotherapy with FOLFIRINOX followed by GnP reported better efficacy with combination therapy [38]. Therefore, for patients with PDAC with low fECV, initiating treatment with the GnP regimen to reduce the degree of tumor microenvironment fibrosis, followed by the more toxic FOLFIRINOX regimen, may yield better efficacy.

Most patients with PDAC present with lymph node metastasis (LNM) at diagnosis [39]. In our study cohort, 59.7% of patients had preoperatively confirmed LNM. Multiple studies, including our investigation, have identified LNM as a significant risk factor for poor postoperative prognosis in PDAC [40, 41, 42, 43]. Therefore, neoadjuvant chemotherapy (NAC) represents a viable option for patients with confirmed preoperative LNM. The role of NAC for patients with resectable pancreatic cancer remains a subject of debate [44], primarily due to the risk of patients becoming ineligible for surgery as a result of disease progression or chemotherapy‐related toxicity [44]. A prospective multicenter clinical trial demonstrated that preoperative chemotherapy with the GnP regimen significantly improved mOS compared to upfront surgery (25.5 months [95% CI, 19.7–29.7 months] vs. 16.7 months [95% CI, 11.6–22.2 months]) [45]. For patients undergoing NAC, non‐invasive assessment using fECV may aid in identifying suitable candidates likely to benefit from the GnP regimen, thereby enabling precision treatment. In conclusion, the results of this study suggest that using fECV to assess the TME of PDAC provides potential insights for the selection of chemotherapy regimens.

To predict the prognosis of PDAC, current research has focused on either using imaging parameters or pathology‐based TME biomarkers alone for prognostic analysis. Our study analyzed the correlation between imaging‐based fECV and pathological indicators α‐SMA and FAP with the clinicopathological characteristics of PDAC patients undergoing postoperative chemotherapy. Notably, fECV, α‐SMA, and FAP were combined for prognostic analysis. Subgroup analysis was conducted for α‐SMA combined with FAP and α‐SMA combined with fECV. In the α‐SMA + FAP group, patients with co‐expression of α‐SMA and FAP had the worst OS, whereas those with co‐expression of low levels of α‐SMA and FAP exhibited significantly better OS. Similarly, in the α‐SMA + fECV group, patients with high α‐SMA and low fECV had significantly shorter OS than those with low α‐SMA and high fECV. Therefore, we believe that these factors synergistically predict the prognosis of patients with PDAC after adjuvant chemotherapy.

## 5 Conclusion

In this study cohort, analysis of the fECV independently measured by the two physicians showed a high ICC, indicating good stability in the fECV measurements. The predictive model established by combining imaging fECV with the pathological indicators α‐SMA and FAP in this study cohort outperformed the models that use only one type of data (imaging, TME biomarkers, or clinicopathological characteristics). In addition, this model combined with three TME biomarkers exhibited better generalization. Therefore, the integration of fECV with α‐SMA and FAP expressions offers a robust method for predicting clinical outcomes in PDAC patients, potentially guiding treatment strategies.

## Author Contributions

L.L., H.Y., W.L. conceived this study. L.L., X.H. performed the experiments. L.L., P.M., X.H. analyzed data. S.X., Q.Z., J.R. queried the patient follow‐up. L.L. wrote the manuscript and prepared all figures. H.Y., Z.Z., X.H. reviewed the manuscript. Y.P. provided the tissue samples. L.L., S.X., P.M. performed the pathology and image evaluation. W.L., H.Y. provided the research facilities.

## Ethics Statement

The study was in accordance with protocols approved by Medical Ethics Committee, Sun Yat‐Sen Memorial Hospital, Sun Yat‐sen University, China (ID: SYS17‐KY‐KS‐2020‐223). This study received approval from the institutional review committee, and the requirement for informed consent was waived.

## Consent

All authors confirm their consent for publication the manuscript.

## Conflicts of Interest

The authors declare no conflicts of interest.

## Supporting information


**Appendix S1:** cam471281‐sup‐0001‐Supinfo.zip.

## Data Availability

The data that support the findings of this study are available from the corresponding author upon reasonable request.
