# Supplementary material for: Extracellular Volume Fraction Combined With Pathological Features of α‐SMA and FAP for Predicting the Prognosis of Patients With Pancreatic Ductal Adenocarcinoma After Surgery and Evaluating the Efficacy of Chemotherapy
Source: Cancer Med. 2025 Oct 2;14(19):e71281. doi: 10.1002/cam4.71281 (PMC12489548; doi:10.1002/cam4.71281)
Supplement: Supplementary file 1 — Appendix S1: cam471281‐sup‐0001‐Supinfo.zip. [file CAM4-14-e71281-s001.zip › cam471281-sup-0003-FigureS1-S2@Supplementary Figures and Legends.docx]

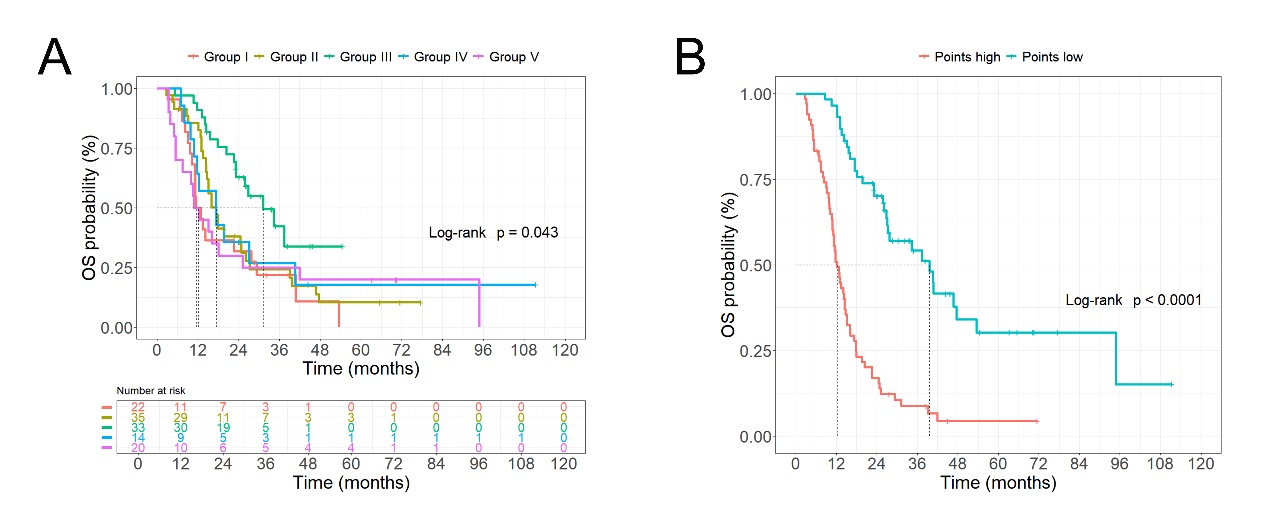


**Fig. S1** K–M survival curve of different chemotherapy regimens and nomogram score. **A**, K–M survival curve of postoperative OS of the study cohort (n = 124) with different chemotherapy regimens. Chemotherapy regimens of group I-IV are FOLFIRINOX, GS, GnP, gemcitabine alone and S-1 alone. **B**, Kaplan–Meier survival curve for the high-score and low-score groups determined by the nomogram.


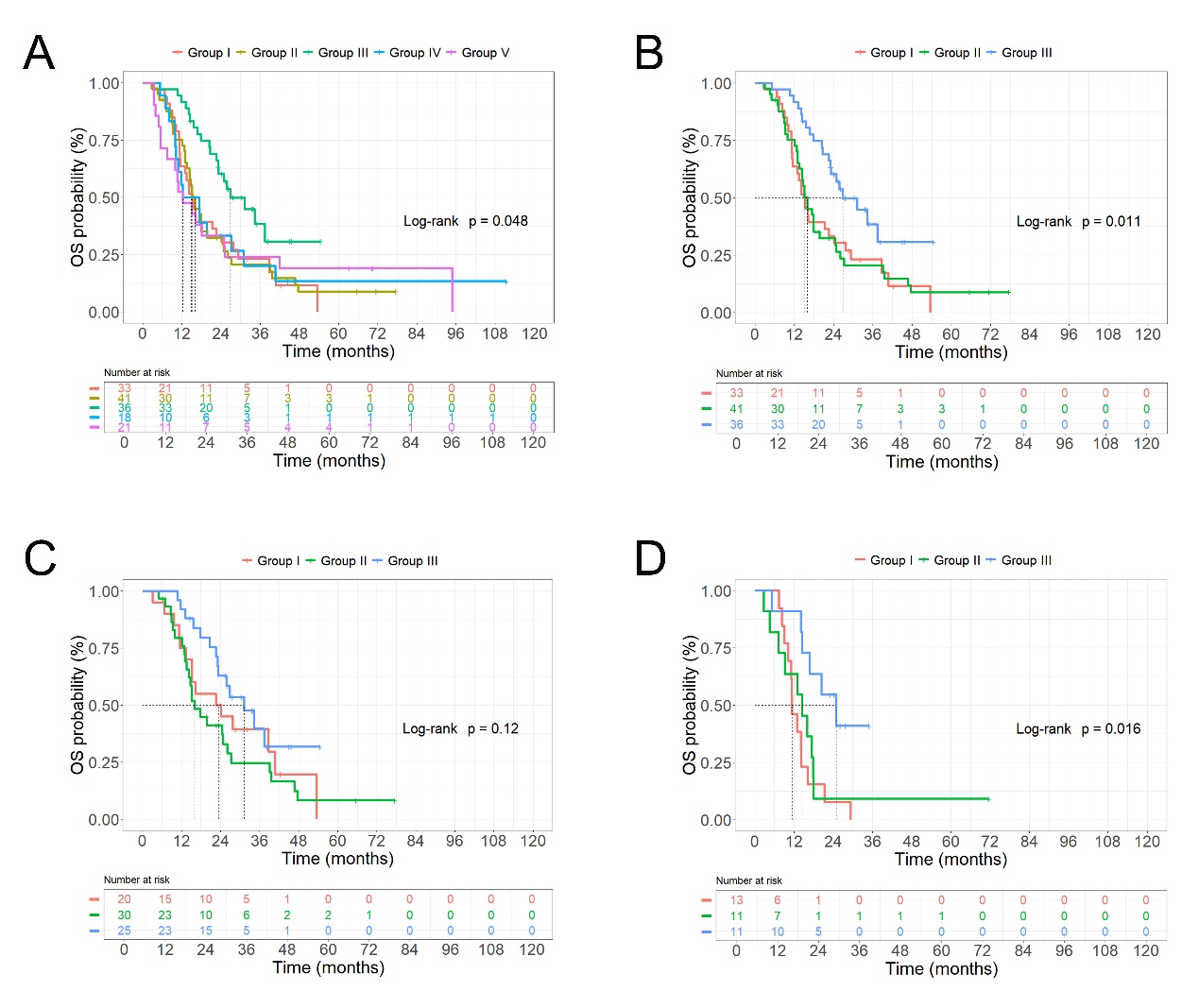


**Figure. S2** K–M survival curves of postoperative OS of the expanded study cohort (n = 149) with different chemotherapy regimens. **A**, K–M curve of the expanded study cohort. Chemotherapy regimens of group I-IV are FOLFIRINOX, GS, GnP, gemcitabine alone and S-1 alone. mOS of each group are 12.2 months, 16.2 months, 25.8 months, 17.4 months, 11.6 months, respectively. **B**, K–M survival curve of the patients with combination chemotherapy regimens. Chemotherapy regimens of group I-III are FOLFIRINOX, GS and GnP. **C-D**, K–M survival curves of patients with combination chemotherapy regimens in high fECV group (**C**) and low fECV group (**D**).
