# Supplementary material for: Extracellular Volume Fraction Combined With Pathological Features of α‐SMA and FAP for Predicting the Prognosis of Patients With Pancreatic Ductal Adenocarcinoma After Surgery and Evaluating the Efficacy of Chemotherapy
Source: Cancer Med. 2025 Oct 2;14(19):e71281. doi: 10.1002/cam4.71281 (PMC12489548; doi:10.1002/cam4.71281)
Supplement: Supplementary file 1 — Appendix S1: cam471281‐sup‐0001‐Supinfo.zip. [file CAM4-14-e71281-s001.zip › cam471281-sup-0004-TableS1-S5@Supplementary Text and Table.docx]

**Supplementary materials**

**Establishment of the study cohort and data collection**

This retrospective study included patients who had undergone surgical intervention for PDAC at the Sun Yat-sen Memorial Hospital of Sun Yat-sen University between January 2014 and December 2021. This study was approved by the institutional review committee, and the requirement for informed consent was waived. The inclusion criteria were as follows: (1) postoperative pathological confirmation of PDAC, with sufficient paraffin-embedded tissue blocks available for experimentation; (2) no prior anticancer treatments, such as chemotherapy, targeted therapy, immunotherapy, or radiotherapy, before surgery; (3) receipt of adjuvant chemotherapy only after surgery; (4) availability of complete imaging, pathological, and clinical data, as well as follow-up information; and (5) negative surgical margins (R0 resection). Patients who met the following criteria were excluded: (1) perioperative death; (2) history of other malignancies; (3) severe organ dysfunction, such as cardiac or pulmonary insufficiency; and (4) history of pancreatitis (related to pancreatic tissue fibrosis). Ultimately, 124 patients met the inclusion criteria and were included in the final study cohort.

The clinical and biochemical information, including sex, age, hematocrit within 3 days of CT examination, CA19-9, CEA, and CA12-5 levels, postoperative pathological tissue differentiation, vascular invasion, perineural invasion, tumor size, location, and lymph node metastasis on contrast-enhanced CT images within 1 week before surgery, were collected. Using clinical data, TNM staging was performed according to the American Joint Committee Cancer (AJCC) staging system (eighth edition).

**Chemotherapy regimens for patients and efficacy assessment of treatment**

1. Gemcitabine plus nab-paclitaxel (GnP) (n=33) (gemcitabine, administered intravenously at 1000 mg/m2; nab-paclitaxel, administered intravenously at 125 mg/m2; once weekly for 3 weeks, followed by 1 week of rest).
2. Gemcitabine plus S-1 (GS) (n=35) (gemcitabine, administered intravenously at 1000 mg/m2 on days 1 and 8; S-1, administered orally at 40 mg/m2 on days 1–14; every 3 weeks).
3. Combination of 5-fluorouracil, leucovorin, irinotecan, and oxaliplatin (FOLFIRINOX) (n = 22) (5-FU, administered intravenously at 2400 mg/m2; leucovorin, administered intravenously at 200 mg/m2; irinotecan, administered intravenously at 150 mg/m2; oxaliplatin, administered intravenously at 85 mg/m2; every 2 weeks).
4. S-1 alone (n = 5) (administered orally at 80 mg/m2 in divided doses twice daily for 4 weeks, followed by 2 weeks of rest).
5. Gemcitabine alone (n = 3) (administered intravenously at 1000 mg/m2 once weekly for 3 weeks, followed by 1 week of rest).

All patients continued to receive the above treatments until tumor progression or serious complications occurred. Patients underwent CT every 3 months postoperatively to evaluate the effectiveness of chemotherapy and tumor progression. The primary endpoint during follow-up was OS, which was defined as the duration from the date of surgery to the time of death from any cause. For patients lost to follow-up, the last recorded follow-up date was considered the endpoint for analysis. For patients who were alive at the time of analysis, the last follow-up date was considered the endpoint. The secondary endpoint was DFS, defined as the duration from the date of surgery to the date of the CT scan, which indicated tumor recurrence, metastasis, or death from any cause. The last follow-up date for all patients was January 2024.

**Immunohistochemistry (IHC) and Multiplex immunohistochemistry (mIHC) staining protocol**

The 4 μm formalin-fixed paraffin-embedded (FFPE) tissue sections were deparaffinized followed by antigen retrieval using Tris-EDTA antigen retrieval solution (pH 9.0) under high temperature and pressure. Subsequently, endogenous peroxidase activity was blocked using hydrogen peroxide for 10 minutes and the sections were incubated in goat serum for 30 minutes to block nonspecific antibody binding. The corresponding primary antibody was then incubated with the sections at 4°C for 16 hours. Afterward, the sections were incubated with horseradish peroxidase (HRP)-conjugated secondary antibody, followed by DAB staining and counterstaining with hematoxylin. Following staining completion, sections were dehydrated stepwise and mounted with neutral resin. Finally, slide scanning was performed using a panoramic slide scanner.

A five-color fluorescence staining kit utilizing tyramide signal amplification (TSA) (Absin Bioscience, abs50013) was employed following the manufacturer's protocol for multiplex immunohistochemistry analysis. The procedure included standard steps such as deparaffinization, hydration, antigen retrieval, quenching of endogenous peroxidase activity, and serum blocking, similar to protocols used in IHC. Tissue sections were incubated with each primary antibodies (refer to Table S4) in 1 hour. Subsequently, secondary antibodies and TSA solutions were applied, utilizing TSA dyes emitting at wavelengths of 480, 520, 570, 620, and 690 for staining. After the final TSA staining, nuclei were counterstained with DAPI for 5 minutes. Slides were sealed with anti-fluorescence quenching agent and panoramic fluorescence images were acquired using the Vectra Polaris fully automated quantitative pathology imaging system instrument (Akoya Biosciences).

**Masson's trichrome staining protocol**

Staining was performed using self-prepared reagents according to the modified Masson method.

Reagent Preparation: Celestine Blue Solution (1.25 g celestine blue, 1.25 g ammonium ferric sulfate, 200 ml distilled water, 30 ml glycerol); Mayer's Hematoxylin Solution (0.1 g hematoxylin, 100 ml distilled water, 5 g potassium alum, 0.1 g citric acid, 20 mg chloral hydrate); Ponceau-Acid Fuchsin Solution (0.7 g Ponceau 2R, 0.3 g acid fuchsin, 99 ml distilled water, 1 ml glacial acetic acid; 2% Aniline Blue Solution: 2 g aniline blue, 98 ml distilled water, 2 ml glacial acetic acid); Bouin’s Solution (75 ml saturated picric acid solution (1.22%), 25 ml formalin, 5 ml glacial acetic acid); 1% Glacial Acetic Acid; 1% Phosphomolybdic Acid.

The staining procedure involves deparaffinizing and rehydrating tissue sections to water. The sections are then mordanted overnight at room temperature in Bouin’s solution or for 2 hours at 37°C, followed by rinsing until the yellow color dissipates. Subsequent steps include staining with celestine blue for 3 minutes and rinsing with water, staining with Mayer's hematoxylin for 3 minutes and rinsing again. Differentiation in 1% acid alcohol follows, with a 10-minute tap water rinse. The sections are then stained with Ponceau-acid fuchsin for 10 minutes, rinsed in distilled water, and treated with 1% phosphomolybdic acid for approximately 10 minutes before direct staining with 2% aniline blue for 5 minutes. A 2-minute treatment with 1% glacial acetic acid solution is followed by rapid dehydration in 95% ethanol and absolute ethanol (I, II, III) for 10 seconds each. Clearing in xylene (I, II, III) for 2 minutes each precedes mounting with neutral resin.

**Analysis of IHC and Masson's trichrome staining**

The tumor-stained slides were scanned using a whole-slide digital scanning system (iViewer). For each slide, five fields of view(1.32 mm × 1.32 mm = 1.74 mm² per field; total ~8.7 mm² per stain) within the tumor lesion were randomly selected by a pathologist with 6 years of experience, avoiding areas containing large blood vessels; subsequent analysis of stained slides was performed following parameter optimization in ImageJ software. The stained area (area) and integral optical density (IOD) were quantified using ImageJ software. The average optical density (AOD) was computed using the formula AOD = IOD/area. AOD was included as an evaluation indicator for IHC staining in the statistical analyses (Figure 1).

Masson's trichrome staining was used to quantify the area occupied by blue collagen fibers, and the collagen volume fraction (CVF) was calculated as the percentage of the collagen fiber area relative to the total analyzed area. The detailed steps of Masson's trichrome staining are provided in the Supplementary Materials.

**Additional CT imaging technology**

The ΔHU^tumor^ is the absolute enhancement of attenuation values between equilibrium and non-enhanced scans of pancreatic cancer. The ΔHU^aorta^ is the absolute enhancement of attenuation values between equilibrium scans and non-enhanced scans of the abdominal aorta at the same level of pancreatic cancer lesions. Necrotic tissue, artifacts, and calcifications should be avoided during ROI selection. Tumor necrosis is characterized by irregular regions with low attenuation on enhanced CT scans. No obvious enhancement in all three phases of contrast-enhanced CT (with a difference in attenuation values between the non-enhanced and enhanced CT ≤10 HU), and the CT attenuation value is ≤20 HU in each phase is indicative of necrosis, and ROIs should be selected from areas with less tumor necrosis. When selecting ROIs for the abdominal aorta, the vessel walls and calcified plaques should be avoided.

**Details of statistical analysis**

Clinicopathological characteristics were examined using either the chi-square test or Fisher’s exact test to determine the relationship between categorical variables and the levels of α-SMA, FAP, and fECV. For continuous variables, differences between groups were analyzed using either the Kruskal–Wallis H test or the Mann–Whitney U test. Pearson or Spearman correlation analyses were conducted to assess the correlation between variables. The consistency of the imaging results analyzed by the different physicians was assessed using the kappa test. The Kaplan–Meier (K–M) method was used to plot the postoperative OS and DFS curves. Survival disparities between the groups were assessed using the log-rank test, and false discovery rate correction of p-values was applied to assess distinctions across multiple groups of K–M survival curves.

A Cox proportional hazards regression model was used to develop a predictive model. Backward elimination was applied with a significance threshold of *p* = 0.05 to identify variables for inclusion in the multivariable Cox regression analysis. The results of the multivariable Cox regression analysis are presented in a forest plot that includes hazard ratios (HR) and 95% confidence intervals (CI) of the p-values. A nomogram was developed for prognostic prediction. The prediction accuracy of the model was evaluated using the receiver operating characteristic (ROC) curves. Additionally, tenfold cross-validation was performed to validate the model. Calibration and decision curve analysis (DCA) curves were plotted to assess the performance of the predictive model. A two-sided *p* < 0.05 indicated statistical significance. All data analyses were performed using SPSS 26.0 (IBM Corp., Armonk, NY, USA) and R software 4.3.2 (R Core Team, Vienna, Austria).

| **Table S1. Clinicopathological characteristics of PDAC research cohorts.** | |
| --- | --- |
| **Clinical Parameters** | **All patient (%)** |
| **Total** | 124 (100.0) |
|  |  |
| **Age(y)** |  |
| ≤60 | 62 (50.0) |
| ＞60 | 62 (50.0) |
| **Gender** |  |
| Male | 68 (54.8) |
| Female | 56 (45.2) |
| **Hypertension** |  |
| Yes | 26 (21.0) |
| No | 98 (79.0) |
| **Diabetes** |  |
| Yes | 26 (21.0) |
| No | 98 (79.0) |
| **CA 19-9 (U/ml)** |  |
| ≤300 | 59 (47.6) |
| ＞300 | 65 (52.4) |
| **CEA (ng/ml)** |  |
| ≤5 | 79 (63.7) |
| ＞5 | 45 (36.3) |
| **CA 12-5 (U/ml)** |  |
| ≤35 | 93 (75.0) |
| ＞35 | 31 (25.0) |
| **Bile duct dilatation** |  |
| Yes | 63 (50.8) |
| No | 61 (49.2) |
| **Pancreatic duct dilatation** |  |
| Yes | 79 (63.7) |
| No | 45 (36.3) |
| **Location** |  |
| Head/Neck | 94 (75.8) |
| Body/tail | 30 (24.2) |
| **AJCC prognostic stage** |  |
| I | 31 (25) |
| II | 53 (42.7) |
| III | 34 (27.4) |
| IV | 6 (4.8) |
| **T stage** |  |
| T1 | 9 (7.3) |
| T2 | 64 (51.6) |
| T3 | 39 (31.5) |
| T4 | 12 (9.7) |
| **N stage** |  |
| N0 | 50 (40.3) |
| N1 | 47 (37.9) |
| N2 | 27 (21.8) |
| **M stage** |  |
| M0 | 108 (86.3) |
| M1 | 17 (13.7) |
| **Histological differentiation** |  |
| Well | 9 (7.3) |
| Moderate | 69 (55.6) |
| Poor | 46 (37.1) |
| **Vascular invasion** |  |
| Yes | 21 (16.9) |
| No | 103 (83.1) |
| **Perineural invasion** |  |
| Yes | 16 (12.9) |
| No | 108 (87.1) |
| **Adjuvant Chemotherapy** |  |
| Gemcitabine alone | 14 (11.3) |
| Tegafur alone | 20 (16.1) |
| Gemcitabine + nab-paclitaxel (AG) | 33 (26.6) |
| Gemcitabine + tegafur (GS) | 35 (28.2) |
| FOLFIRINOX (FFX) | 22 (17.7) |

| **Table S2. Cutoff values for fECV, FAP and α-SMA** | |
| --- | --- |
| **Clinical Parameters** | **All patient (%)** |
| **Extracellular volume fraction** |  |
| High | 80 (64.5) |
| Low | 44 (35.5) |
| **FAP AOD** |  |
| High | 41 (33.1) |
| Low | 83 (66.9) |
| **α-SMA AOD** |  |
| High | 97 (78.2) |
| Low | 27 (21.8) |

**Table S3. Multiple Cox proportional hazards regression models.**

| Model | Cox regression model variables |
| --- | --- |
| Model 1 | clinicopathological characteristics + α-SMA + FAP + fECV |
| Model 2 | clinicopathological characteristics + α-SMA + FAP |
| Model 3 | clinicopathological characteristics + fECV |
| Model 4 | clinicopathological characteristics + FAP + fECV |
| Model 5 | clinicopathological characteristics |

**Table S4. Average AUC of tenfold cross-validation and the AUDC for each model**

| Model | Mean AUC of k Fold CV | Area Under DCA Curve | | | |
| --- | --- | --- | --- | --- | --- |
|  |  | 1-year OS | 2-year OS | 3-year OS | Median OS |
| Model 1 | 0.847 | 0.104 | 0.245 | 0.301 | 0.206 |
| Model 2 | 0.783 | 0.084 | 0.228 | 0.294 | 0.187 |
| Model 3 | 0.748 | 0.060 | 0.191 | 0.258 | 0.160 |
| Model 4 | 0.776 | 0.073 | 0.181 | 0.200 | 0.156 |
| Model 5 | 0.747 | 0.047 | 0.151 | 0.213 | 0.127 |

| **Table S5. Information on the primary antibodies used in IHC and their incubation message.** | | |
| --- | --- | --- |
| Antibody | Group | Message |
| Mouse monoclonal [1A4] to alpha smooth muscle Actin | Abcam Plc（#ab7817） | 1:200  16h at 4℃ |
| Rabbit monoclonal [EPR20021] to Fibroblast activation protein, alpha | Abcam Plc（#ab207178） | 1:200  16h at 4℃ |
