# Supplementary figures and images for: Extracellular Volume Fraction Combined With Pathological Features of α‐SMA and FAP for Predicting the Prognosis of Patients With Pancreatic Ductal Adenocarcinoma After Surgery and Evaluating the Efficacy of Chemotherapy
Source: Cancer Med. 2025 Oct 2;14(19):e71281. doi: 10.1002/cam4.71281 (PMC12489548; doi:10.1002/cam4.71281)

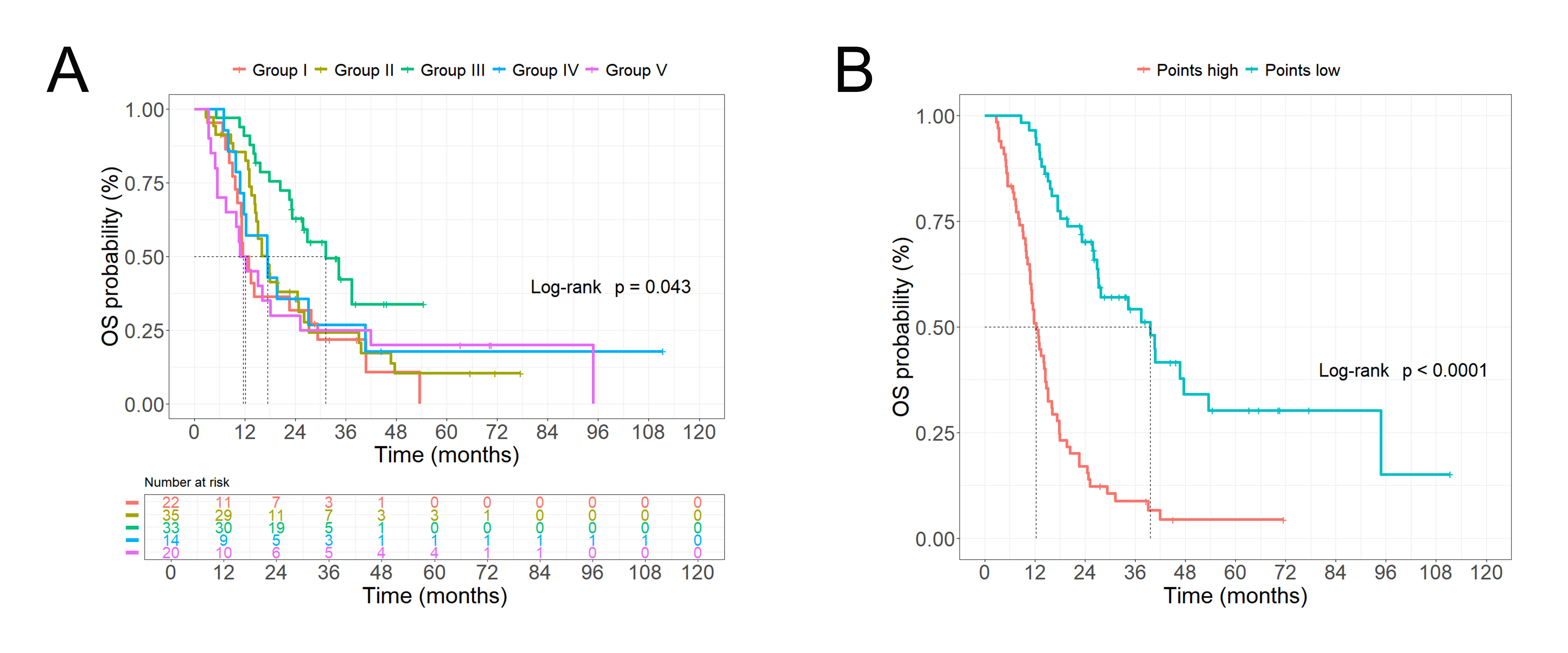

Supplement: Supplementary file 1 — Appendix S1: cam471281‐sup‐0001‐Supinfo.zip. [file CAM4-14-e71281-s001.zip › cam471281-sup-0001-Supinfo1@figure s1.jpg]

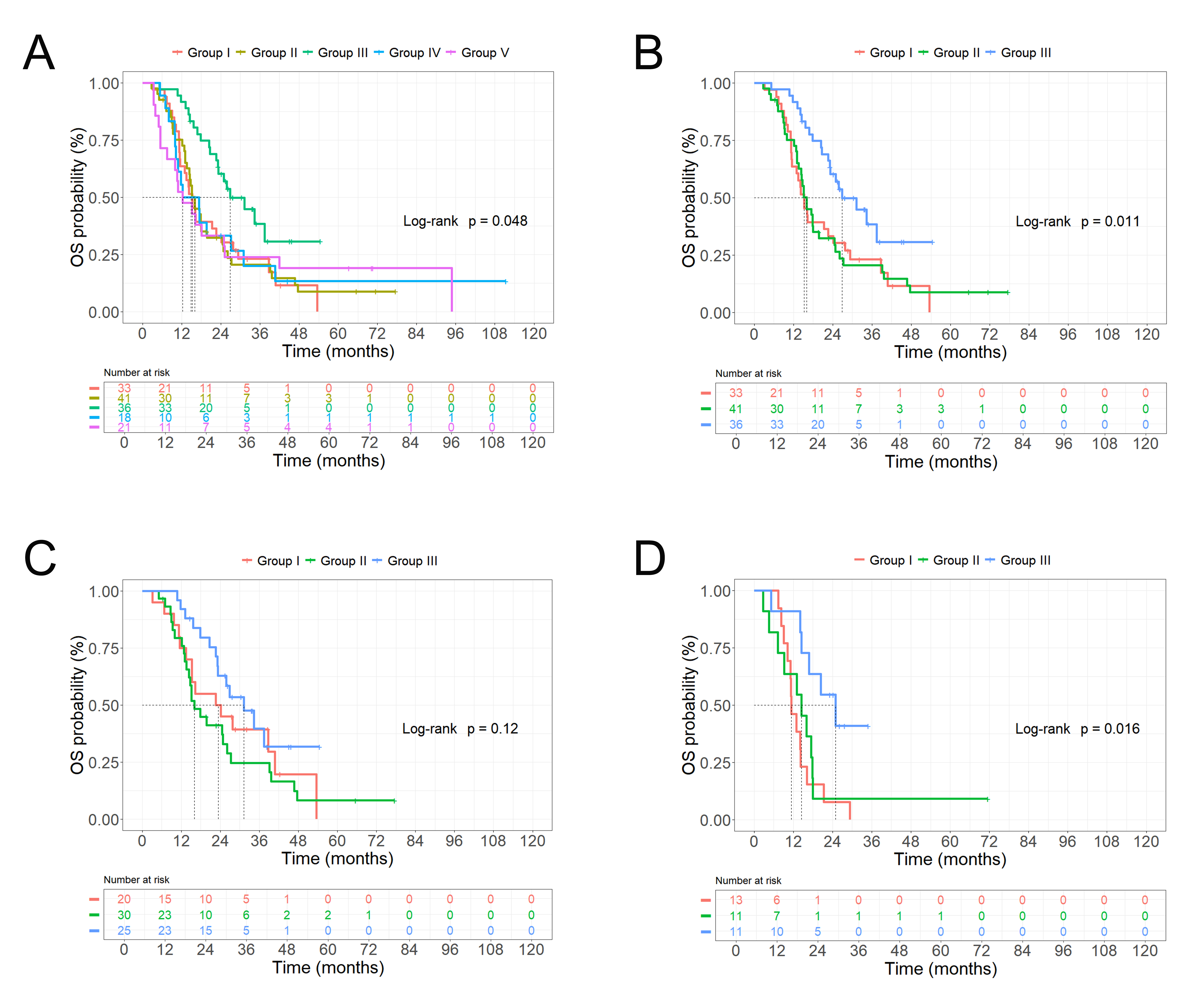

Supplement: Supplementary file 1 — Appendix S1: cam471281‐sup‐0001‐Supinfo.zip. [file CAM4-14-e71281-s001.zip › cam471281-sup-0002-Supinfo2@figure s2.jpg]
